# Association of increased participation in social activity in later life with risk of all-cause mortality and heart diseases in older people: results from the Chinese Longitudinal Healthy Longevity Survey (CLHLS)

**DOI:** 10.3389/fpubh.2024.1396184

**Published:** 2024-06-25

**Authors:** Ziqiong Wang, Changchun Chen, Haiyan Ruan, Sen He

**Affiliations:** ^1^Department of Cardiology, West China Hospital of Sichuan University, Chengdu, China; ^2^Department of Cardiology, Hospital of Traditional Chinese Medicine, Chengdu, China; ^3^Karamay Hospital of Integrated Chinese and Western Medicine, Xinjiang, China

**Keywords:** all-cause mortality, change, heart diseases, older people, social activity

## Abstract

**Background:**

Previous studies have shown social activity is associated with reduced risk of health outcomes. However, among older people (≥65 years) who were socially inactive at baseline, limited study explored whether increased participation in social activity in later life was associated with reduced risk of health outcomes; therefore, using the data from the Chinese Longitudinal Healthy Longevity Survey, the study was performed.

**Methods:**

The study outcomes were 10-year all-cause mortality (sample number = 9,984) and 10-year heart diseases (sample number = 7,496). The exposure was the change of social activity frequency. Cox regression analysis was used for data analysis.

**Results:**

During the follow-up, there were 6,407 all-cause mortalities and 1,035 heart diseases, respectively. Kaplan–Meier analysis demonstrated that cumulative incidences of all-cause mortality were significantly lower in participants with changes into more frequent social activity (log-rank *p* < 0.001), while no significant difference was observed for heart diseases (log-rank *p* = 0.330). Compared with the subgroup who never participated in social activity at baseline, adjusted HRs of all-cause mortality were 0.79 (95% CI: 0.70–0.90, *p* < 0.001), 0.78 (95% CI: 0.63–0.96, *p* = 0.019), 0.74 (0.59–0.92, *p* = 0.006), and 0.70 (95% CI: 0.56–0.88, *p* = 0.002) for the subgroup of switching to sometimes, the subgroup of switching to once a month, the subgroup of switching to once a week, and the subgroup of switching to everyday, respectively. The corresponding HRs of heart diseases were 0.83 (95% CI: 0.65–1.08, *p* = 0.170), 0.82 (95% CI: 0.51–1.31, *p* = 0.412), 0.91 (0.58–1.42, *p* = 0.675) and 0.75 (95% CI: 0.47–1.20, *p* = 0.227), respectively. Stratified and sensitivity analyses revealed similar results.

**Conclusion:**

Among older people who never participated in social activity, increased participation in social activity in later life was associated with reduced risk of all-cause mortality, but was not associated with reduced risk of heart diseases.

## 1 Introduction

Population aging has become a major medical and socio-demographic issue worldwide due to the increased lifespan and reduced fertility rate (1, 2). Previous critical aging research has focused on multidimensional fields for health promotion in older people, such as addressing the effect of socioeconomic status in relation to the acceleration of aging (3), delineating the association between lifestyle behaviors and healthy aging (4), demonstrating the potential influence of psychological factors on biological aging (5), as well as exploring some other important internal and external factors that contribute to healthy aging (6), in order to achieve a holistically successful aging through both personal and social-level interventions.

For social factors related to successful aging, a previous study has categorized them into four aspects, including social engagement/participation, social support, social integration/network, and socio-demographic/socioeconomic factors (7). Of which, social participation means that person’s involvement in activities providing interactions with others in society or the community. For social activities in social participation, there are six levels of involvement of the individual with others: (1) doing an activity in preparation for connecting with others, (2) being with others, (3) interacting with others without doing a specific activity with them, (4) doing an activity with others, (5) helping others, and (6) contributing to society (8). Previous studies have shown that active social participation is particularly a protective determinant in reducing overall mortality risk in older people (9, 10), as well as decreasing the risk of physical frailty and contributing to better sleep quality in older people (11, 12). Even in older frail people, active social participation could achieve clinical benefits in reducing the risk of subsequent disability and mortality (13). Participation in organized social activities, namely performing activities with others (the 4th level), is the most direct and representative way of social participation. In our recently published study, we have demonstrated that daily participation in organized social activity is associated with long-term survival in older Chinese people (14). However, important gaps remain. First, social activity participation is a dynamic process, which can change over time (15). To the author’s knowledge, most studies used information from baseline surveys and assumed the frequency of social participation remained unchanged during the follow-up (9, 10, 13). Only one study had assessed the impact of the changes in the frequency of social participation on all-cause mortality and indicated no significant health benefits of initiated social participation in later life (16). Therefore, among older people who never participate in social activity at baseline, it is largely unknown whether increased participation in social activity in later life is associated with reduced risk of health outcomes. Second, previous results regarding the effect of social participation on heart diseases were inconsistent, some of which indicated protective effect (17), while others indicated neutral effect (18–20) in middle aged and older population or general population. Still, for older people, the impact of increased participation in social activity on the new onset of heart diseases remains to be investigated.

Therefore, among older people who never participated in social activity at baseline, we conducted an analysis to assess the impact of increased participation in social activity in later life on the risk of 10-year all-cause mortality as well as the risk of 10-year heart diseases, using the data obtained from the Chinese Longitudinal Healthy Longevity Survey (CLHLS). The study may provide more valuable information about social activity recommendation in older people.

## 2 Methods

### 2.1 Study participants

The CLHLS is an ongoing, prospective and nationwide cohort study of community-dwelling Chinese older people. The survey aims to better understand the determinants of healthy longevity among older people aged over 65 years old. The survey is carried out in a randomly chosen half of the counties and cities in 23 of the 31 provinces, which covers roughly 85% of the Chinese population. Trained interviewers conduct the survey in the homes of the participants. The survey started in 1998, with subsequent follow-up in 2000, 2002, 2005, 2008, 2011, 2014, and 2018. New participants are enrolled during the following waves from 1998 to reduce the attrition due to death and loss to follow-up. More details about CLHLS have been reported elsewhere, and data quality was reported to be generally good (21, 22). The CLHLS was carried out in accordance with the principles of the Declaration of Helsinki, and was authorized by the research Ethics Committee of Peking University (IRB00001052-13074). Before participating, survey respondents provided their informed consent.

**Figure 1:**
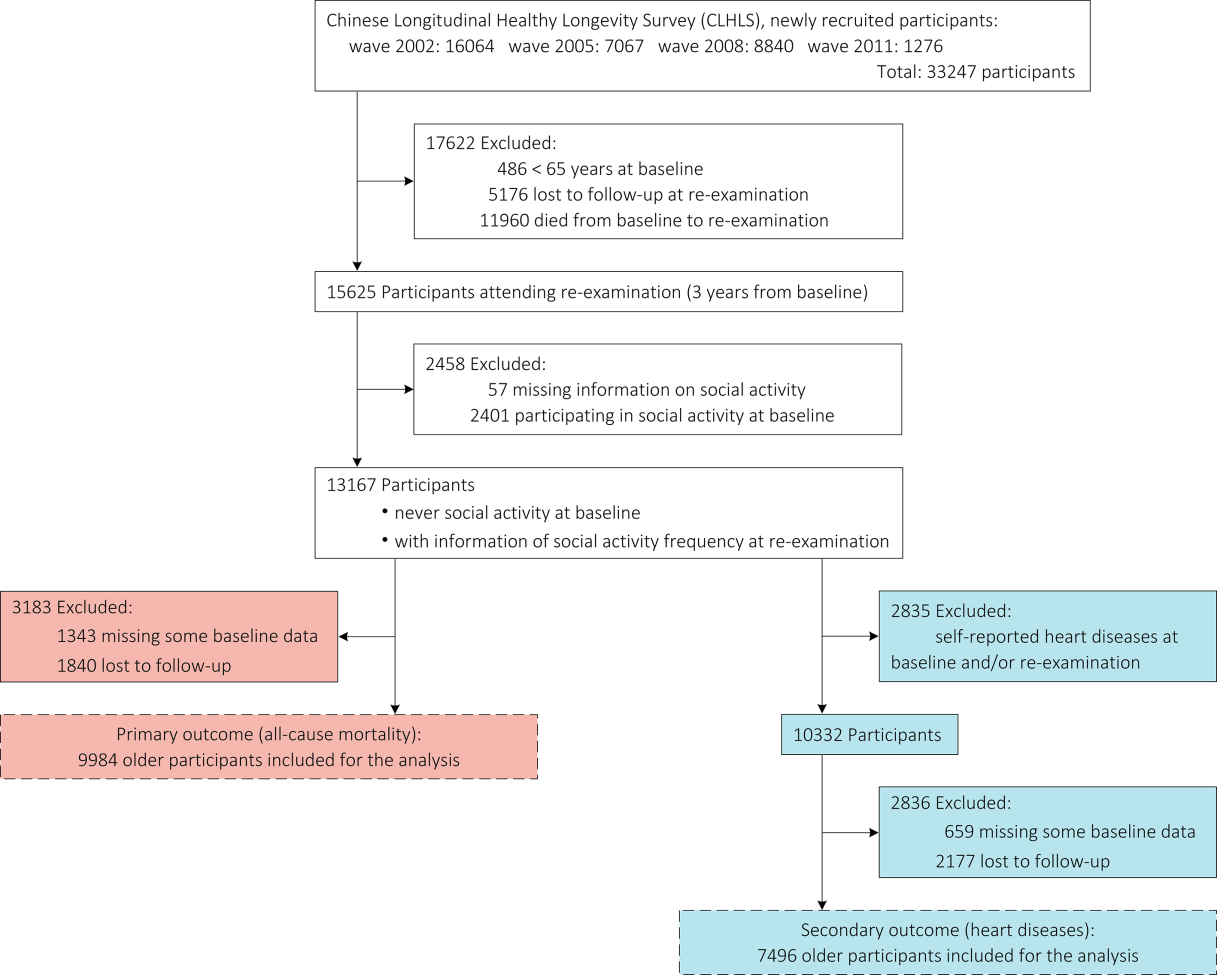
Flow chart.

Figure 1 depicts the detailed recruitment process of study participants. Because social activity was examined since wave 2002, the present study was based on the newly recruited participants since 2002 who completed the examinations of social activity for two consecutive waves (i.e., 2002 and 2005, 2005, and 2008, 2008 and 2011, 2011, and 2014). The final sample consisted of 9,984 older participants for assessing the risk of all-cause mortality and 7,496 older participants for assessing the risk of heart diseases, respectively.

### 2.2 Assessment of social activity

Social activity was assessed by the participation frequency at baseline, which was classified as “1, almost everyday; 2, not daily, but at least once/week; 3, not weekly, but at least once/month; 4, not monthly, but sometimes; 5, never” by the question “Do you take part in some social activities (organized)?.” Social activity in the present study refers to “organized social activity,” which could reflect participants’ positive attitude toward social participation than other daily activities, such as housework, garden work, raising domestic animals.

**Figure 2:**
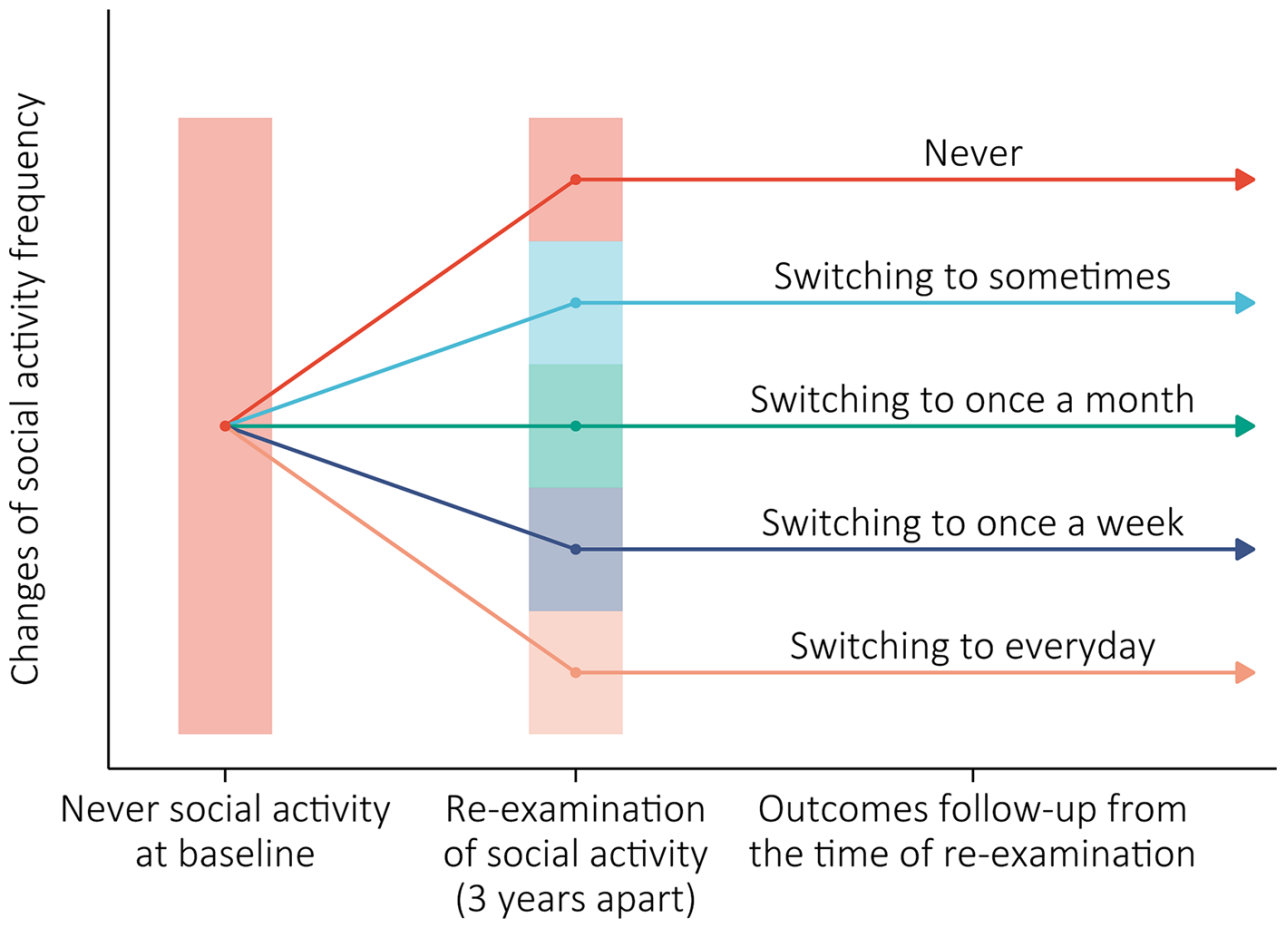
Longitudinal changes of social activity frequency from baseline to re-examination.

Based on the research purposes, the included participants never participated in social activity at baseline, and had complete information of social activity frequency at re-examination. Therefore, five types of longitudinal changes in social activity frequency from baseline to re-examination were determined as: never, switching to sometimes, switching to once a month, switching to once a week, and switching to everyday (Figure 2).

### 2.3 Assessment of other covariates

Supplementary Table 1 shows the detailed information of other covariates, which were obtained through questionnaires, including: sex, age, education, marital status, residence, co-residence, current smoking, current drinking, current regular exercise, regular intake of foods (fruits, vegetables, meats, fishes, eggs, and beans), comorbidities (hypertension, diabetes, heart diseases, cerebrovascular diseases, respiratory diseases, and cancer), and activities of daily living (ADL) disability. More detailed information about these covariates can be found on: https://agingcenter.duke.edu/CLHLS.

### 2.4 Study outcome

The primary outcome was 10-year all-cause mortality, and the secondary outcome was 10-year new onset of heart diseases (ICD codes: I00-I09, I11, I13, and I20-I52). For all-cause mortality, survival status and date of death were collected through interviews with close family members during each survey, and were certified from death certificates, hospital admission records, and medical records if available. In the CLHLS, the reliability of mortality may be more reliable than those obtained from the national census, although some recall errors occurred (23). The follow-up period was the time between re-examination of social activity and either death, loss to follow-up or 10-year period (Figure 2).

The new onset of heart diseases was defined by self-reported history for the survivors, and ascertained from a close family member according to the question “was the participant suffering from heart disease prior to death?” for the deceased interviewees. Follow-up began from the time of re-examination. The CLHLS did not provide the detailed date of new onset of heart diseases. Therefore, based on the literatures (24, 25), time to event was defined as the period from baseline to the earlier interview for those who survived; for those who died, the time from baseline to the date of death was calculated. Censored observations were defined as participants who did not have heart diseases, and the censoring time was calculated from baseline to the last interview or the date of death or the end of the study (10-year period) (Figure 2).

### 2.5 Statistical analysis

We conducted the analyses with the following steps: (1) comparisons of baseline data; (2) evaluating adjusted risk of changes of social activity frequency for all-cause mortality and heart diseases; (3) performing interaction and stratified analyses; and (4) executing sensitivity analyses.

Baseline characteristics were displayed across the five changes of social activity frequency, and the characteristics were described as median (interquartile range, IQR) for continuous variables and number (percentage) for categorical variables. For continuous variables, *p*-value for trend across the five changes was computed from the Pearson test when row-variable was normal distribution and from the Spearman test when it was non-normal distribution. When the row-variable was categorical, *p*-value for trend was computed from Mantel–Haenszel test of trend.

Kaplan–Meier method was used to estimate the cumulative incidence of outcomes in each group, and the log-rank test was used for comparisons. Cox proportional-hazard regression analysis was used to examine the association between changes of social activity frequency and outcomes, with the follow-up time (years) as the time scale, and no evidence of violation of the proportional-hazard assumption was found. Multicollinearity diagnostic tests were performed by variance inflation factor (VIF) before determining the final model, and a VIF greater than 10 is generally considered to indicate statistically significant issue with multicollinearity. The tests showed that the VIF was less than 2.0 for all variables in the final model. In addition, stratified analysis was used to assess the consistency of main findings in various subgroups, and interactions were examined by likelihood ratio testing.

Furthermore, to assess the robustness of main findings, we performed a series of sensitivity analyses, including: (1) to clarify the role of participants lost to follow-up in the associations, we did sensitivity analyses for such participants censored at two time points: median and the end of follow-up; (2) to reduce potential reverse causation, we excluded the deaths within the first year or first 2 years of follow-up; (3) to account for missing data (Supplementary Table 2), we used multiple imputation as another sensitivity analysis; (4) given that this was a observational study, it was necessary to achieve comparability of groups (never vs. switching to social activity) with regard to potential confounding variables, and this was accomplished using propensity score matching (PSM); (5) to account for the competing risk between heart diseases and all-cause mortality, the Fine-Gray model was fitted to assess the association between changes of social activity frequency and heart diseases; (6) to address the confounding effect of age, we conducted a sensitivity analysis using age as the time scale; (7) to address the confounding effect of study sites, we conducted a sensitivity analysis further adjusting provinces; and (8) to assess the potential for unmeasured confounding between changes of social activity frequency and outcomes, we calculated the *E*-values, which quantify the required magnitude of an unmeasured confounder that could negate the observed association between exposures and outcomes.

All analyses were performed with R version 4.1.0 including the “compareGroups,” “survival,” “tidyverse,” “rms,” “mice,” “forestplot,” “survminer,” and “stats” packages.^1^ All tests were two sided, and *p* values <0.05 were considered statistically significant.

## 3 Results

### 3.1 Baseline characteristics

Table 1 shows the baseline characteristics of population used for assessing all-cause mortality. Generally speaking, participants who switched to a more frequent participation of social activity were more likely to be male participants and younger, had higher prevalence of longer education, more likely to be married and live in urban area, had higher prevalence of current smoking, current drinking and current regular exercise, as well as higher prevalence of diabetes and lower prevalence of ADL disability. Other detailed information is presented in Table 1. Baseline characteristics of participants used for assessing risk of incidental heart diseases are shown in Supplementary Table 3.

**Table 1:** Baseline characteristics of population used for assessing all-cause mortality.

	All	Never	Switching to sometimes	Switching to once a month	Switching to once a week	Switching to everyday	*p*-value for trend^a^
No. of participants	9,984	8,943	546	167	151	177	
Sex: Male	4,181 (41.88%)	3,599 (40.24%)	309 (56.59%)	91 (54.49%)	85 (56.29%)	97 (54.80%)	<0.001
Age (years)	84.00 (73.00, 92.00)	85.00 (74.00, 93.00)	77.00 (68.00, 85.00)	75.00 (68.50, 85.50)	80.00 (70.00, 89.00)	74.00 (67.00, 83.00)	<0.001
Education							<0.001
No school	6,372 (63.82%)	5,904 (66.02%)	236 (43.22%)	84 (50.30%)	70 (46.36%)	78 (44.07%)	
1 year or more	3,612 (36.18%)	3,039 (33.98%)	310 (56.78%)	83 (49.70%)	81 (53.64%)	99 (55.93%)	
Marital status							<0.001
Not in marriage	6,123 (61.33%)	5,655 (63.23%)	241 (44.14%)	80 (47.90%)	77 (50.99%)	70 (39.55%)	
In marriage	3,861 (38.67%)	3,288 (36.77%)	305 (55.86%)	87 (52.10%)	74 (49.01%)	107 (60.45%)	
Residence							<0.001
Urban	3,432 (34.38%)	2,943 (32.91%)	267 (48.90%)	66 (39.52%)	69 (45.70%)	87 (49.15%)	
Rural	6,552 (65.62%)	6,000 (67.09%)	279 (51.10%)	101 (60.48%)	82 (54.30%)	90 (50.85%)	
Co-residence							0.069
With family members	8,317 (83.30%)	7,456 (83.37%)	456 (83.52%)	136 (81.44%)	118 (78.15%)	151 (85.31%)	
Alone	1,504 (15.06%)	1,367 (15.29%)	63 (11.54%)	26 (15.57%)	26 (17.22%)	22 (12.43%)	
In an institution	163 (1.63%)	120 (1.34%)	27 (4.95%)	5 (2.99%)	7 (4.64%)	4 (2.26%)	
Current smoking	2082 (20.85%)	1820 (20.35%)	134 (24.54%)	41 (24.55%)	36 (23.84%)	51 (28.81%)	<0.001
Current drinking	2,135 (21.38%)	1860 (20.80%)	148 (27.11%)	41 (24.55%)	38 (25.17%)	48 (27.12%)	0.001
Current regular exercise	2,818 (28.23%)	2,397 (26.80%)	218 (39.93%)	62 (37.13%)	60 (39.74%)	81 (45.76%)	<0.001
Regular intake of foods							
Fruits	3,382 (33.87%)	2,934 (32.81%)	232 (42.49%)	63 (37.72%)	62 (41.06%)	91 (51.41%)	<0.001
Vegetables	8,819 (88.33%)	7,880 (88.11%)	494 (90.48%)	145 (86.83%)	135 (89.40%)	165 (93.22%)	0.042
Meats	4,657 (46.64%)	4,137 (46.26%)	285 (52.20%)	76 (45.51%)	65 (43.05%)	94 (53.11%)	0.112
Fishes	2,622 (26.26%)	2,267 (25.35%)	192 (35.16%)	48 (28.74%)	45 (29.80%)	70 (39.55%)	<0.001
Eggs	5,106 (51.14%)	4,541 (50.78%)	286 (52.38%)	85 (50.90%)	88 (58.28%)	106 (59.89%)	0.005
Beans	4,314 (43.21%)	3,800 (42.49%)	264 (48.35%)	81 (48.50%)	77 (50.99%)	92 (51.98%)	<0.001
Comorbidities							
Hypertension	1,647 (16.50%)	1,474 (16.48%)	100 (18.32%)	22 (13.17%)	21 (13.91%)	30 (16.95%)	0.712
Diabetes	183 (1.83%)	152 (1.70%)	14 (2.56%)	5 (2.99%)	7 (4.64%)	5 (2.82%)	0.004
Heart diseases	753 (7.54%)	667 (7.46%)	46 (8.42%)	17 (10.18%)	6 (3.97%)	17 (9.60%)	0.518
Cerebrovascular diseases	434 (4.35%)	388 (4.34%)	19 (3.48%)	9 (5.39%)	8 (5.30%)	10 (5.65%)	0.394
Respiratory diseases	1,018 (10.20%)	910 (10.18%)	61 (11.17%)	15 (8.98%)	17 (11.26%)	15 (8.47%)	0.775
Cancer	28 (0.28%)	22 (0.25%)	2 (0.37%)	2 (1.20%)	1 (0.66%)	1 (0.56%)	0.051
ADL disability	1,427 (14.29%)	1,354 (15.14%)	34 (6.23%)	18 (10.78%)	13 (8.61%)	8 (4.52%)	<0.001

Values are median (IQR) or *n* (%).

^a^Across the groups: never vs. switching to sometimes vs. switching to once a month vs. switching to once a week vs. switching to everyday.

ADL, activities of daily living; IQR, inter-quartile range.

### 3.2 Association between changes of social activity and outcomes

**Figure 3:**
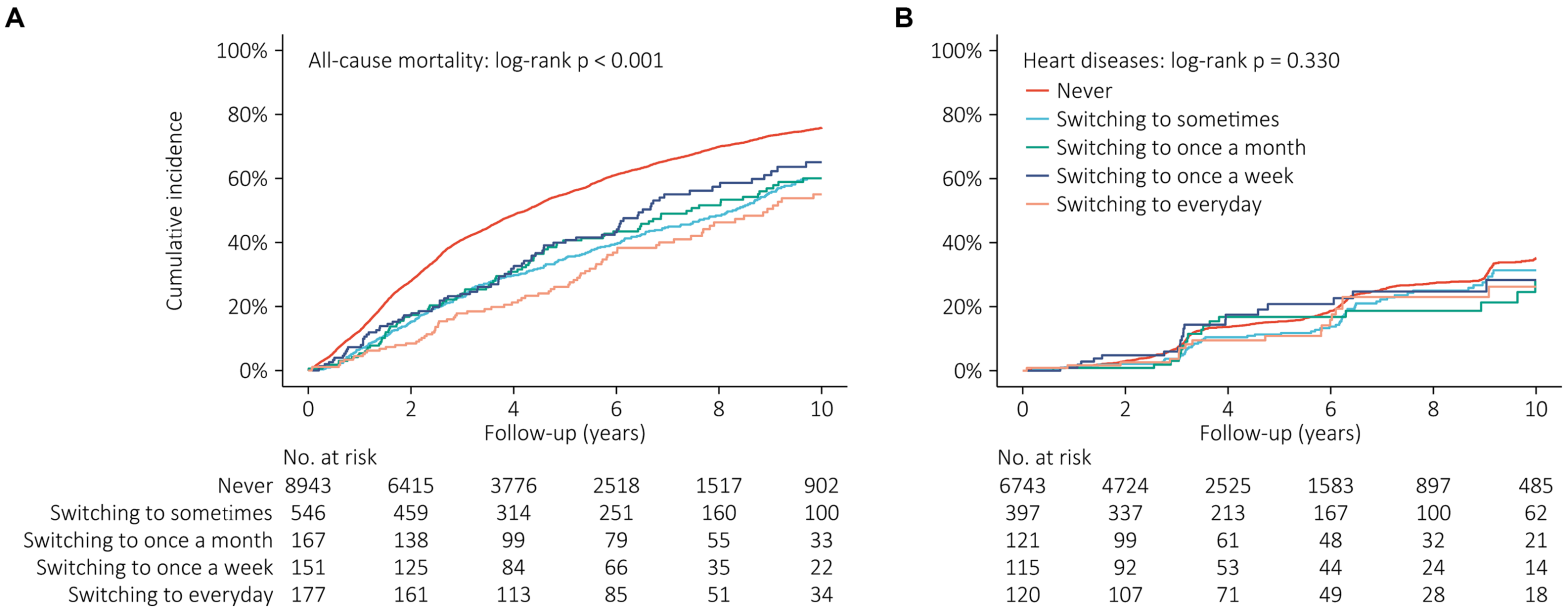
Kaplan–Meier curves showing cumulative incidence of all-cause mortality **(A)** and heart disease **(B)** across different changes of social activity frequency.

There were 6,407 all-cause mortalities during a follow-up of 44,122.7 person-years. Kaplan–Meier analysis demonstrated that the cumulative incidence of all-cause mortality was significantly lower in older people who switched to a more frequent social activity participation (log-rank *p* < 0.001, Figure 3A). There were 1,035 heart diseases during a follow-up of 30,545.4 person-years. However, Kaplan–Meier analysis did not show any significant difference regarding the cumulative incidence of heart disease among these groups (log-rank *p* = 0.330, Figure 3B).

Overall, when compared to subjects who remained never participating in social activity during follow-up time, those who switched to participate in social activity had significantly lower risk of 10-year all-cause mortality with adjusted hazard ratio (HR) at 0.77 (95% CI: 0.70–0.84, *p* < 0.001). While switching of social activity participation failed to exert any clinical benefits in reducing the risk of 10-year heart diseases (adjusted HR: 0.83, 95% CI: 0.68–1.01, *p* = 0.061).

**Figure 4:**
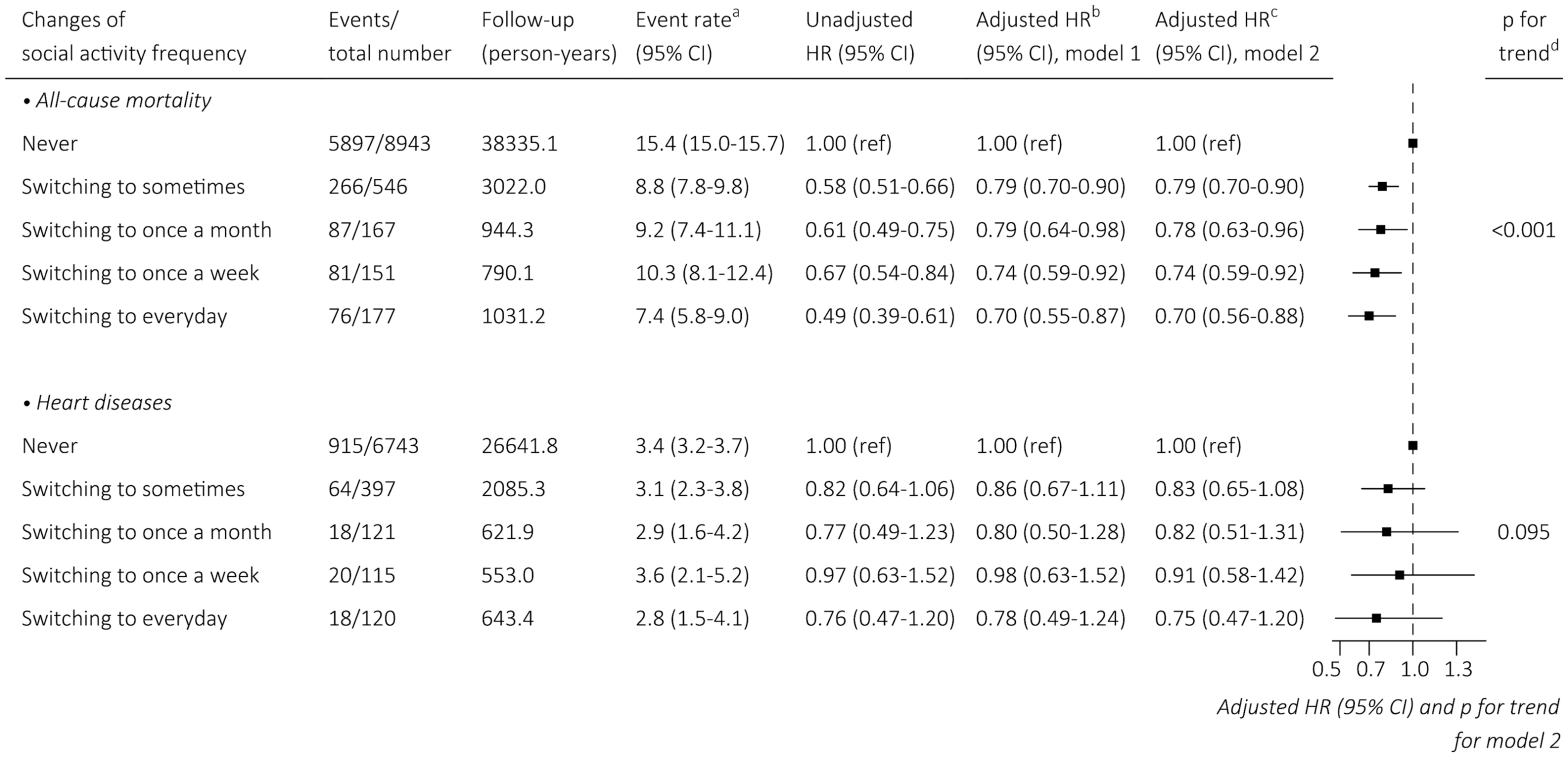
Association between changes of social activity frequency and outcomes. ^a^Per 100 person-years. ^b^Adjustment with sex, and age. ^c^For all-cause mortality: adjustment with sex, age, education, marital status, residence, co-residence, current smoking, current drinking, current regular exercise, regular intake of foods, comorbidities (as shown in Table 1), and ADL disability. For heart diseases: adjustment with sex, age, education, marital status, residence, co-residence, current smoking, current drinking, current regular exercise, regular intake of foods, comorbidities (as shown in Supplementary Table 3), and ADL disability. ^d^The values obtained from Wald tests of a linear association of the score as a numeral (1–5) with the risk of all-cause mortality and heart diseases. CI, confidence interval; HR, hazard ratio.

Compared with the subgroup of remained never participating in social activity during the follow-up time, the multivariate-adjusted HRs of all-cause mortality were 0.79 (95% CI: 0.70–0.90, *p* < 0.001), 0.78 (95% CI: 0.63–0.96, *p* = 0.019), 0.74 (0.59–0.92, *p* = 0.006), and 0.70 (95% CI: 0.56–0.88, *p* = 0.002) in the subgroup of switching to sometimes, subgroup of switching to once a month, subgroup of switching to once a week and subgroup of switching to everyday, which indicated that the risk of 10-year all-cause mortality gradually decreased in subjects who switched to a more frequent social activity participation (*p* value for trend <0.001). The fully adjusted HRs of heart diseases were 0.83 (95% CI: 0.65–1.08, *p* = 0.170), 0.82 (95% CI: 0.51–1.31, *p* = 0.412), 0.91 (0.58–1.42, *p* = 0.675) and 0.75 (95% CI: 0.47–1.20, *p* = 0.227) in the subgroup of switching to sometimes, subgroup of switching to once a month, subgroup of switching to once a week and subgroup of switching to everyday when comparing to the subgroup of remained never participating in social activity during the follow-up time. The corresponding *p* value for trend was 0.095, which indicated no significant impact of social activity participation on the risk of 10-year heart diseases (Figure 4).

### 3.3 Stratified analysis

For all-cause mortality, results of analyses were consistent in the subgroups by sex, education, marital status, co-residence, lifestyles, co-morbidities and ADL disability with all *p*_interaction_ ≥ 0.05 (Supplementary Figure 1). Age (age < 84 years vs. age ≥ 84 years) and residence site (rural vs. urban) were significant interactive factors in the association between changes of frequency in social activity participation and all-cause mortality. The effect of increased participation in social activity on incidental heart diseases was similar across different strata defined by sex, age, education, residence site, co-residence, lifestyles, co-morbidities and ADL disability with all *p*_interaction_ ≥ 0.05 (Supplementary Figure 2). Marital status was a significant interactive term with *p*_interaction_ = 0.019.

### 3.4 Sensitivity analysis

Extensive sensitivity analyses were conducted to support the main findings. When treating participants lost to follow-up as censored at the median or the end of follow-up, the results did not change materially (Supplementary Tables 4, 5). Additionally, the results remained similar after excluding deaths within the first or first 2 years (Supplementary Tables 6, 7). Similar results were also observed after multiple imputation to treat missing data (Supplementary Table 8).

The distribution of PS in the subgroup of remained never participating in social activity and subgroup of switching to social activity participation before matching is shown in Supplementary Figures 3A, 4A. The lesser overlap of PS curves of the two subgroups indicated a greater risk of confounding. After PSM, PS curves for subgroup of remained never participating in social activity and subgroup of switching to social activity participation were superimposed, and also the absolute standardized difference was less than 0.10 for each variable, both of which indicated well-balanced baseline covariates between the two subgroups (Supplementary Figures 3B,C, 4B,C). Still, association between changes of social activity frequency and outcomes in the PSM sample remained the same (Supplementary Table 9).

Cumulative incidence functions suggested that, after adjusted for competing risk of all-cause mortality, the cumulative incidence of heart diseases still showed no significant difference among these subgroups (*p* = 0.544, Supplementary Figure 5). Cox regression analysis demonstrated similar results as well (Supplementary Table 10). When using age as time scale, the results were similar to the main results (Supplementary Table 11). After further adjusting study sites, the results did not change materially either (Supplementary Table 12). For all-cause mortality, based on model 2 in Figure 2, the *E*-values were 1.64, 1.66, 1.76, 1.88 in subgroup of switching to sometimes, subgroup of switching to once a month, subgroup of switching to once a week and subgroup of switching to everyday, respectively. For heart diseases, the corresponding *E*-values were 1.69, 1.74, 1.43, and 1.99 (Supplementary Table 13).

## 4 Discussion

In the present study, our main finding was that when compared to older people who never participated in social activity, those with increased participation in social activity in later life had a significantly lower risk of 10-year all-cause mortality. The more frequent social activity participation as they increased, the lower the risk of 10-year all-cause mortality. However, increase in social activity participation did not show any benefit of reduced risk of 10-year heart diseases in older people. Stratified analyses and in-depth sensitivity analyses supported the robustness of the main findings.

Regarding the frequency of social activity participation as a dynamically changing variable and analyzing its impact on health outcomes, few studies have followed this path before. In a longitudinal study consisted of 2,240 older people aged 60 years and older, and subjects were categorized into none, initiated, decreased and continued pattern based on the changes in the frequency of social participation, Shimatani et al. demonstrated that the continued or decreased pattern was associated with a decreased risk of all-cause mortality, particularly in male subjects. The authors also urged initiation of social participation at an earlier phase of life transition since the initiated pattern failed to show any benefit in reducing mortality risk (16). The results were inconsistent with our findings. With a relatively larger sample size, our study indicated that increased social activity participation in later life could still be able to reduce the risk of 10-year all-cause mortality, and no gender difference was observed. What’s more, there is a dose–response relationship, in which the mortality risk gradually decreased along with gradually increased frequency of social activity participation.

Another interesting finding was that no significant impact of increased participation in social activity on the risk of 10-year heart diseases in older people. In fact, evidence about the association between social participation or social isolation [defined as a state of complete or near-complete lack of contact between an individual and society (26)] and cardiovascular disease is controversial. In a large cohort of 8,422 middle-aged and older subjects, Guo et al. demonstrated that subjects with fluctuating social isolation and consistently high social isolation showed significantly higher risk of incident cardiovascular disease when compared to those with consistently low social isolation (17). Conversely, in a previous study consisted of 33,538 middle-aged Japanese, Oshio et al. reported that social participation had no impact in preventing the onset of heart disease (18). Additionally, in another longitudinal analysis using data from the English Longitudinal Study of Aging, Bu et al. demonstrated that there was little evidence that social isolation was independently associated with the risk of either cardiovascular disease diagnosis or admission (19). Some other study may report a significant association between social isolation and incident heart failure in general population, however, this association was potentially modified by subjective loneliness status (20). Those results suggest that maybe the subjective aspects of social connections are more important in relation to heart diseases in comparison to the objective status of social participation or social isolation.

Although identifying the reasons to account for the healthy beneficial effects of social activity participation is beyond the scope of the present study, some possible explanations have been discussed in our recently published article (14). Dropping risky health behaviors (e.g., smoking) and engaging in beneficial health behaviors (e.g., physical activity, healthy dietary habitats), and contributing to the phycological well-beings may mediate the association (27–31). Considering the positive impact of social activity participation on overall health in older people, it is extremely essential to make efforts to motivate social activity participation in older people facing the aging population. A number of studies have shed light on factors determining the social participation in older people. Some individual factors, such as levels of education, socioeconomic status, disability, physical factors (e.g., having urinary incontinence, having pain, impaired endurance, using a mobility device) and cost, as well as some environmental factors, such as social networks with families or friends, social support and access barriers (e.g., transportation issues) (32–35), seemed to be related to social participation of older people. Therefore, the promotion of social activity participation in older people should focus primarily on those vulnerable individuals, especially those with health and/or social obstacles.

Our study has a number of strengths, including dynamic evaluation of the frequency of social activity participation, in-depth analysis about the association between switching of social activity participation and heart disease in addition to all-cause mortality, and extensive sensitivity analyses to support the main findings. Nevertheless, some limitations need to be acknowledged. Firstly, the frequency of social participation before the baseline survey was unknown. Participants participated in social activity before the time of baseline survey but stopped social activity participation may classified into subgroup of never, leading to underestimated HRs. Secondly, we mainly focused on the changes of social activity frequency, while the types of social activity in which the participants participated in were not considered. Thirdly, the reasons for the changes of frequency of social activity participation were not established in the present study. Fourthly, for the statistical analysis, although many confounders have been adjusted, still some other residual confounding factors were not adjusted for. Fifthly, the use of self-reported data on heart diseases and some other covariates may lead to biased results. It would be hard to address the measurement error considering the nature of the epidemiological study. Lastly, survivorship bias might affect the strength of the null finding about incident heart diseases. However, the information about incident heart disease was also collected from their relatives of deceased interviewees. Besides, the association between changes of social activity frequency and incident heart disease, accounting for competing risk of all-cause mortality has also been conducted. Those maneuverers could help to reduce the survivorship bias. Future research warrants longitudinal studies that addressing those limitations to provide more useful information about the social activity participation in relation to health outcomes.

## 5 Conclusion

Among older people who never participated in social activity at baseline, increased social activity in later life was associated with reduced risk of all-cause mortality, and a gradient of association between social activity frequency and the risk of 10-year all-cause mortality was found. However, increased social activity in later life was not associated with reduced risk of heart diseases. On the whole, increased participation in social activity in later life is beneficial to health outcomes, and these findings suggest that developing public health policies to motivate older people to increase participating in social activity should be urged for overall health promotion.

## Data availability statement

Publicly available datasets were analyzed in this study. This data can be found at: https://agingcenter.duke.edu/CLHLS.

## Ethics statement

The studies involving humans were approved by the CLHLS was carried out in accordance with the principles of the Declaration of Helsinki, and was authorized by the research Ethics Committee of Peking University (IRB00001052-13074). Before participating, survey respondents provided their informed consent. The studies were conducted in accordance with the local legislation and institutional requirements. The participants provided their written informed consent to participate in this study.

## Author contributions

ZW: Conceptualization, Data curation, Methodology, Writing – original draft, Writing – review & editing. CC: Data curation, Methodology, Writing – original draft. HR: Data curation, Formal analysis, Writing – original draft. SH: Conceptualization, Formal analysis, Funding acquisition, Methodology, Writing – review & editing.

## Footnotes

^1^ http://www.R-project.org
