# Supplementary material for: Association of increased participation in social activity in later life with risk of all-cause mortality and heart diseases in older people: results from the Chinese Longitudinal Healthy Longevity Survey (CLHLS)
Source: Front Public Health. 2024 Jun 25;12:1396184. doi: 10.3389/fpubh.2024.1396184 (PMC11231370; doi:10.3389/fpubh.2024.1396184)

Association of increased participation in social activity in later life with risk of all-cause mortality and heart diseases in older people: results from the Chinese Longitudinal Healthy Longevity Survey (CLHLS)

Ziqiong Wang[1], MD, PhD, Changchun Chen[1], RGN, Haiyan Ruan[1, 2], MD, Sen He[1,3] *, MD, PhD

1. Department of Cardiology, West China Hospital of Sichuan University, Chengdu, China.
2. Department of Cardiology, Hospital of Traditional Chinese Medicine, Shuangliu District, Chengdu, China.
3. Karamay Hospital of Integrated Chinese and Western Medicine, Xinjiang Uygur Autonomous Region, China.

*Corresponding author: Sen He; Department of Cardiology, West China Hospital of Sichuan University, No.37 Guoxue Alley, Chengdu, China; Email: hesen_sky@scu.edu.cn

List of Supplementary Materials

Supplementary Table 1. Definitions of baseline variables in the present study 3
Supplementary Table 2. Distributions of baseline variables with missing data 6
Supplementary Table 3. Baseline characteristics of participants used for assessing risk of heart diseases 7
Supplementary Table 4. Association between changes of social activity frequency and outcomes, in considering the losses censored at the median of follow-up (3.37 years for all-cause mortality, and 3.19 for heart diseases years, respectively) 9
Supplementary Table 5. Association between changes of social activity frequency and outcomes, in considering the losses censored at the end of follow-up (10 years for all-cause mortality and heart diseases) 10
Supplementary Table 6. Association between changes of social activity frequency and outcomes, after excluding deaths within the first year 11
Supplementary Table 7. Association between changes of social activity frequency and outcomes, after excluding deaths within the first two years 12
Supplementary Table 8. Association between changes of social activity frequency and outcomes after multiple imputationa 13
Supplementary Table 9. Association between changes of social activity frequency and outcomes in the PSM sample[a] 14
Supplementary Table 10. Association between changes of social activity frequency and incidental heart diseases, accounting for competing risk of all-cause mortality 15
Supplementary Table 11. Association between changes of social activity frequency and outcomes, with age as the time scale 16
Supplementary Table 12. Association between changes of social activity frequency and outcomes, further controlling provinces 17
Supplementary Table 13. E-value assessing unmeasured confoundings between changes of social activity frequency and outcomes ... 18
Supplementary Figure 1. Stratified analyses by potential modifiers of the association between changes of social activity frequency and all-cause mortality 19
Supplementary Figure 2. Stratified analyses by potential modifiers of the association between changes of social activity frequency and heart diseases 20
Supplementary Figure 3. Propensity score distributional overlap and ASD (for assessing risk of all-cause mortality) 21
Supplementary Figure 4. Propensity score distributional overlap and ASD (for assessing risk of heart diseases) 22
Supplementary Figure 5. Cumulative incidence of heart diseases based on changes of social activity frequency (solid line) and adjusted for competing risk of all-cause mortality 22

Supplementary Table 1. Definitions of baseline variables in the present study

Variables in the present study	Components of variable	Questions in the CLHLS questionnaire	Options in the CLHLS questionnaire	Scales of reclassification in the present study
Sex			• male<br>• female	• Male<br>• Female
Age				• Continuous (years)
Education		How many years did you attend school?	• years of school<br>• don't know<br>• missing	• No school: years of school = 0<br>• 1 year or more: years of school >= 1<br>• missing: don't know, missing
Marital status		Current marital status?	• currently married and living with spouse<br>• separated<br>• divorced<br>• widowed<br>• never married<br>• don't know<br>• missing	• In marriage: currently married and living with spouse, separated<br>• Not in marriage: divorced, widowed, never married<br>• missing: don't know, missing
Residence		Current residence area of interviewee?	• city<br>• town<br>• rural	• Urban: city, town<br>• Rural: rural
Co-residence		Co-residence?	• with household member(s)<br>• alone<br>• in an institution<br>• missing	• With family members: with household member(s)<br>• Alone: alone<br>• In an institution: in an institution<br>• missing: missing
Current smoking		Do you smoke at present?	• yes<br>• no<br>• missing	• Yes: yes<br>• No: no<br>• missing: missing
Current drinking		Do you drink at present?	• yes<br>• no<br>• missing	• Yes: yes<br>• No: no<br>• missing: missing
Current regular exercise		Do you do exercises regularly at present?	• yes<br>• no<br>• don't know<br>• missing	• Current regular exercise: yes<br>• No regular exercise at present: no<br>• missing: don't know, missing
Regular intake of fruits, and vegetables, respectively		Do you eat these foods, respectively?	• almost everyday<br>• except winter/quite often<br>• occasionally<br>• rarely or never<br>• don't know<br>• missing	• Regular intake: almost everyday, except winter/quite often<br>• No regular intake: occasionally, rarely or never<br>• missing: don't know, missing
Regular intake of meats, fishes, eggs, and beans, respectively		Do you eat these foods, respectively?	Waves 2002, 2005<br>• almost everyday<br>• occasionally	• Regular intake: almost everyday; not everyday, but at least once per week<br>• No regular intake: not every week, but at least

			• rarely or never<br>• missing<br><br>Waves 2008, 2011<br>• almost everyday<br>• not everyday, but at least once per week<br>• not every week, but at least once per month<br>• not every month, but occasionally<br>• rarely or never<br>• don't know<br>• missing	once per month; not every month, but occasionally; occasionally; rarely or never.<br>• missing: don't know, missing
Hypertension, diabetes, heart diseases, cerebrovascular diseases, respiratory diseases, and cancer, respectively		Are you suffering from these diseases, respectively?	• yes<br>• no<br>• don't know<br>• missing	• Yes: yes<br>• No: no<br>• missing: don't know, missing
ADL disability	Bathing	Without assistance?	• without assistance<br>• one part assistance<br>• more than one part assistance<br>• don't know<br>• missing	• In the CLHLS survey, six items of daily self-care ability were collected from each participant based on the Katz index: dressing, bathing, transferring, toileting, continence, and eating. Each item included three answers: complete independence, partially dependence, and complete dependence.<br>ADL disability was defined as present if participants needed any assistance in performing at least one of the six self-care activities.<br>• missing: don't know, missing
	Dressing	Get clothes and get completely dressed without assistance?	• without assistance<br>• need assistance for trying shoes<br>• assistance in getting clothes and getting dressed<br>• missing	
	Toileting	Go to the toilet, cleans self, and arranges clothes without assistance (may use object for support such as cane, walker, or wheelchair)?	• without assistance<br>• assistance in cleaning or arranging clothes<br>• don't use toilet<br>• missing	
	Transferring	Get in and out of bed as well as in and out of a chair without assistance (may use object for support such as cane or walker)?	• without assistance<br>• with assistance<br>• bedridden<br>• missing	
	Continence	Has complete control of urination and bowel movement without assistance?	• without assistance<br>• occasional accidents<br>• incontinent<br>• missing	
	Feeding	Feed self without assistance?	• without assistance<br>• with some help<br>• need feeding<br>• missing	

More detailed information about these covariates can be found on: https://agingcenter.duke.edu/CLHLS.
Abbreviations: ADL = activities of daily living, CLHLS = Chinese Longitudinal Healthy Longevity Surveys.

Supplementary Table 2. Distributions of baseline variables with missing data

Variables	Participants used for assessing all-cause mortality		Participants used for assessing heart diseases	
	Number of missing data	Percentage of missing data (%)	Number of missing data	Percentage of missing data (%)
Sex	0	0.00	0	0.00
Age	0	0.00	0	0.00
Education	50	0.38	40	0.39
Marital status	5	0.04	4	0.04
Residence	0	0.00	0	0.00
Co-residence	12	0.09	9	0.09
Current smoking	9	0.07	6	0.06
Current drinking	11	0.08	8	0.08
Current regular exercise	28	0.21	19	0.18
Regular intake of foods				
Fruits	3	0.02	3	0.03
Vegetables	13	0.10	12	0.12
Meats	5	0.04	5	0.05
Fishes	5	0.04	5	0.05
Eggs	4	0.03	3	0.03
Beans	4	0.03	4	0.04
Comorbidities				
Hypertension	476	3.62	110	1.06
Diabetes	496	3.77	61	0.59
Heart diseases	484	3.68	NA	NA
Cerebrovascular diseases	457	3.47	40	0.39
Respiratory diseases	411	3.12	52	0.50
Cancer	592	4.50	145	1.40
ADL disability	334	2.54	260	2.52

Participants with missing values were deleted in the main statistical analyses, and we performed multiple imputation for missing values as a sensitivity analysis.
Abbreviations: ADL = activities of daily living.

Supplementary Table 3. Baseline characteristics of participants used for assessing risk of heart diseases

Variables	All	Never	Switching to sometimes	Switching to once a month	Switching to once a week	Switching to everyday	p value for trend[a]
No. of participants	7496	6743	397	121	115	120	
Sex: male	3164 (42.21%)	2745 (40.71%)	224 (56.42%)	64 (52.89%)	62 (53.91%)	69 (57.50%)	<0.001
Age (years)	85.00 (74.00, 93.00)	86.00 (74.00, 93.00)	76.00 (68.00, 85.00)	76.00 (70.00, 86.00)	81.00 (70.00, 89.50)	75.00 (67.00, 84.00)	<0.001
Education							<0.001
No school	4884 (65.15%)	4533 (67.23%)	177 (44.58%)	67 (55.37%)	57 (49.57%)	50 (41.67%)	
1 year or more	2612 (34.85%)	2210 (32.77%)	220 (55.42%)	54 (44.63%)	58 (50.43%)	70 (58.33%)	
Marital status							<0.001
Not in marriage	4700 (62.70%)	4351 (64.53%)	175 (44.08%)	60 (49.59%)	63 (54.78%)	51 (42.50%)	
In marriage	2796 (37.30%)	2392 (35.47%)	222 (55.92%)	61 (50.41%)	52 (45.22%)	69 (57.50%)	
Residence							<0.001
Urban	2452 (32.71%)	2128 (31.56%)	176 (44.33%)	42 (34.71%)	51 (44.35%)	55 (45.83%)	
Rural	5044 (67.29%)	4615 (68.44%)	221 (55.67%)	79 (65.29%)	64 (55.65%)	65 (54.17%)	
Co-residence							0.110
With family members	6221 (82.99%)	5598 (83.02%)	333 (83.88%)	98 (80.99%)	89 (77.39%)	103 (85.83%)	
Alone	1152 (15.37%)	1057 (15.68%)	41 (10.33%)	19 (15.70%)	21 (18.26%)	14 (11.67%)	
In an institution	123 (1.64%)	88 (1.31%)	23 (5.79%)	4 (3.31%)	5 (4.35%)	3 (2.50%)	
Current smoking	1602 (21.37%)	1406 (20.85%)	106 (26.70%)	26 (21.49%)	25 (21.74%)	39 (32.50%)	0.002
Current drinking	1663 (22.19%)	1465 (21.73%)	109 (27.46%)	30 (24.79%)	24 (20.87%)	35 (29.17%)	0.023
Current regular exercise	2068 (27.59%)	1782 (26.43%)	141 (35.52%)	46 (38.02%)	44 (38.26%)	55 (45.83%)	<0.001
Regular intake of foods							
Fruits	2473 (32.99%)	2156 (31.97%)	164 (41.31%)	45 (37.19%)	47 (40.87%)	61 (50.83%)	<0.001
Vegetables	6599 (88.03%)	5921 (87.81%)	360 (90.68%)	106 (87.60%)	102 (88.70%)	110 (91.67%)	0.131
Meats	3464 (46.21%)	3099 (45.96%)	204 (51.39%)	57 (47.11%)	46 (40.00%)	58 (48.33%)	0.700
Fishes	1962 (26.17%)	1711 (25.37%)	137 (34.51%)	34 (28.10%)	33 (28.70%)	47 (39.17%)	<0.001
Eggs	3773 (50.33%)	3386 (50.22%)	198 (49.87%)	58 (47.93%)	63 (54.78%)	68 (56.67%)	0.201
Beans	3204 (42.74%)	2846 (42.21%)	181 (45.59%)	58 (47.93%)	55 (47.83%)	64 (53.33%)	0.002
Comorbidities							
Hypertension	1033 (13.78%)	930 (13.79%)	62 (15.62%)	14 (11.57%)	13 (11.30%)	14 (11.67%)	0.415
Diabetes	96 (1.28%)	79 (1.17%)	10 (2.52%)	2 (1.65%)	4 (3.48%)	1 (0.83%)	0.093
Cerebrovascular diseases	271 (3.62%)	246 (3.65%)	11 (2.77%)	5 (4.13%)	4 (3.48%)	5 (4.17%)	0.995
Respiratory diseases	727 (9.70%)	658 (9.76%)	41 (10.33%)	10 (8.26%)	11 (9.57%)	7 (5.83%)	0.257
Cancer	18 (0.24%)	16 (0.24%)	1 (0.25%)	0 (0.00%)	0 (0.00%)	1 (0.83%)	0.611
ADL disability	1057 (14.10%)	1013 (15.02%)	19 (4.79%)	11 (9.09%)	11 (9.57%)	3 (2.50%)	<0.001

Values are median (IQR) or n (%).

[a] Across the groups: never vs. switching to sometimes vs. switching to once a month vs. switching to once a week vs. switching to everyday.
Abbreviations: ADL = activities of daily living, IQR = inter-quartile range.

Supplementary Table 4. Association between changes of social activity frequency and outcomes, in considering the losses censored at the median of follow-up (3.37 years for all-cause mortality, and 3.19 for heart diseases years, respectively)

	Never	Switching to sometimes	Switching to once a month	Switching to once a week	Switching to everyday
All-cause mortality					
No. of participants (n)	10545	660	210	191	218
Events (n)	5937	267	87	81	77
Follow-up (person-years)	54089.9	4155.3	1374.3	1190.1	1434.6
Event rate (95% CI)[a]	11.0 (10.7-11.2)	6.4 (5.7-7.2)	6.3 (5.0-7.6)	6.8 (5.4-8.2)	5.4 (4.2-6.5)
Adjusted HR (95% CI)[b], p	1.00 (ref)	0.84 (0.74-0.95), 0.005	0.76 (0.62-0.94), 0.013	0.77 (0.62-0.97), 0.023	0.74 (0.59-0.93), 0.009
Heart diseases					
No. of participants (n)	8643	526	168	159	177
Events (n)	920	65	18	20	18
Follow-up (person-years)	44309.8	3299.1	1065.1	993.0	1184.7
Event rate (95% CI)[a]	2.1 (1.9-2.2)	2.0 (1.5-2.4)	1.7 (0.9-2.5)	2.0 (1.1-2.9)	1.5 (0.8-2.2)
Adjusted HR (95% CI)[b], p	1.00 (ref)	0.91 (0.70-1.17), 0.461	0.76 (0.47-1.21), 0.251	0.92 (0.59-1.43), 0.706	0.71 (0.44-1.13), 0.151

[a] per 100 person-years.

[b] For all-cause mortality: adjustment with sex, age, education, marital status, residence, co-residence, current smoking, current drinking, current regular exercise, regular intake of foods, comorbidities (as shown in Table 1), and ADL disability. For heart diseases: adjustment with sex, age, education, marital status, residence, co-residence, current smoking, current drinking, current regular exercise, regular intake of foods, comorbidities (as shown in eTable 3), and ADL disability.

Abbreviations: CI = confidence interval, HR = hazard ratio.

Supplementary Table 5. Association between changes of social activity frequency and outcomes, in considering the losses censored at the end of follow-up (10 years for all-cause mortality and heart diseases)

	Never	Switching to sometimes	Switching to once a month	Switching to once a week	Switching to everyday
All-cause mortality					
No. of participants (n)	10545	660	210	191	218
Events (n)	5937	267	87	81	77
Follow-up (person-years)	54355.1	4162.0	1374.3	1190.1	1441.2
Event rate (95% CI)[a]	10.9 (10.7-11.2)	6.4 (5.7-7.2)	6.3 (5-7.6)	6.8 (5.4-8.2)	5.3 (4.2-6.5)
Adjusted HR (95% CI)[b], p	1.00 (ref)	0.84 (0.74-0.95), 0.005	0.77 (0.62-0.95), 0.014	0.78 (0.62-0.97), 0.027	0.74 (0.59-0.92), 0.008
Heart diseases					
No. of participants (n)	8643	526	168	159	177
Events (n)	920	65	18	20	18
Follow-up (person-years)	44752.4	3312.7	1078.7	993.0	1191.5
Event rate (95% CI)[a]	2.1 (1.9-2.2)	2.0 (1.5-2.4)	1.7 (0.9-2.4)	2.0 (1.1-2.9)	1.5 (0.8-2.2)
Adjusted HR (95% CI)[b], p	1.00 (ref)	0.91 (0.71-1.17), 0.469	0.75 (0.47-1.20), 0.236	0.92 (0.59-1.44), 0.731	0.71 (0.44-1.13), 0.151

[a] per 100 person-years.

[b] For all-cause mortality: adjustment with sex, age, education, marital status, residence, co-residence, current smoking, current drinking, current regular exercise, regular intake of foods, comorbidities (as shown in Table 1), and ADL disability. For heart diseases: adjustment with sex, age, education, marital status, residence, co-residence, current smoking, current drinking, current regular exercise, regular intake of foods, comorbidities (as shown in eTable 3), and ADL disability.

Abbreviations: CI = confidence interval, HR = hazard ratio.

Supplementary Table 6. Association between changes of social activity frequency and outcomes, after excluding deaths within the first year

	Never	Switching to sometimes	Switching to once a month	Switching to once a week	Switching to everyday
All-cause mortality					
No. of participants (n)	7837	510	158	140	169
Events (n)	4791	230	78	70	68
Follow-up (person-years)	37761.4	2997.8	938.9	784.3	1026.8
Event rate (95% CI)[a]	12.7 (12.4-13.0)	7.7 (6.7-8.6)	8.3 (6.5-10.1)	8.9 (6.9-10.9)	6.6 (5.1-8.1)
Adjusted HR (95% CI)[b], p	1.00 (ref)	0.80 (0.70-0.92), 0.001	0.80 (0.64-1.01), 0.057	0.73 (0.58-0.93), 0.010	0.71 (0.56-0.90), 0.005
Heart diseases					
No. of participants (n)	6676	393	120	114	118
Events (n)	848	60	17	19	16
Follow-up (person-years)	26606.8	2083.2	621.3	552.3	642.4
Event rate (95% CI)[a]	3.2 (3.0-3.4)	2.9 (2.2-3.6)	2.7 (1.5-4.0)	3.4 (1.9-5.0)	2.5 (1.3-3.7)
Adjusted HR (95% CI)[b], p	1.00 (ref)	0.82 (0.63-1.07), 0.137	0.81 (0.50-1.31), 0.385	0.90 (0.57-1.43), 0.666	0.69 (0.42-1.14), 0.150

[a] per 100 person-years.
[b] For all-cause mortality: adjustment with sex, age, education, marital status, residence, co-residence, current smoking, current drinking, current regular exercise, regular intake of foods, comorbidities (as shown in Table 1), and ADL disability. For heart diseases: adjustment with sex, age, education, marital status, residence, co-residence, current smoking, current drinking, current regular exercise, regular intake of foods, comorbidities (as shown in eTable 3), and ADL disability.
Abbreviations: CI = confidence interval, HR = hazard ratio.

Supplementary Table 7. Association between changes of social activity frequency and outcomes, after excluding deaths within the first two years

	Never	Switching to sometimes	Switching to once a month	Switching to once a week	Switching to everyday
All-cause mortality					
No. of participants (n)	6443	463	138	125	162
Events (n)	3397	183	58	55	61
Follow-up (person-years)	35723.0	2927.0	908.5	763.9	1017.0
Event rate (95% CI)[a]	9.5 (9.2-9.8)	6.3 (5.4-7.1)	6.4 (4.8-8.0)	7.2 (5.4-9.0)	6 (4.5-7.5)
Adjusted HR (95% CI)[b], p	1.00 (ref)	0.82 (0.70-0.95), 0.009	0.75 (0.58-0.97), 0.031	0.72 (0.55-0.94), 0.014	0.78 (0.61-1.01), 0.060
Heart diseases					
No. of participants (n)	6573	389	120	110	117
Events (n)	745	56	17	15	15
Follow-up (person-years)	26448.8	2077.3	621.3	547.1	640.5
Event rate (95% CI)[a]	2.8 (2.6-3.0)	2.7 (2.0-3.4)	2.7 (1.5-4.0)	2.7 (1.4-4.1)	2.3 (1.2-3.5)
Adjusted HR (95% CI)[b], p	1.00 (ref)	0.82 (0.63-1.09), 0.168	0.88 (0.54-1.43), 0.616	0.78 (0.47-1.31), 0.355	0.71 (0.42-1.18), 0.185

[a] per 100 person-years.

[b] For all-cause mortality: adjustment with sex, age, education, marital status, residence, co-residence, current smoking, current drinking, current regular exercise, regular intake of foods, comorbidities (as shown in Table 1), and ADL disability. For heart diseases: adjustment with sex, age, education, marital status, residence, co-residence, current smoking, current drinking, current regular exercise, regular intake of foods, comorbidities (as shown in eTable 3), and ADL disability.

Abbreviations: CI = confidence interval, HR = hazard ratio.

Supplementary Table 8. Association between changes of social activity frequency and outcomes after multiple imputation[a]

	Never	Switching to sometimes	Switching to once a month	Switching to once a week	Switching to everyday
All-cause mortality					
No. of participants (n)	9981	585	182	164	192
Events (n)	6660	287	95	89	83
Follow-up (person-years)	42390.8	3243.5	1037.7	856.0	1109.4
Event rate (95% CI)[b]	15.7 (15.4-16.1)	8.8 (7.9-9.8)	9.2 (7.4-10.9)	10.4 (8.4-12.4)	7.5 (5.9-9.0)
Adjusted HR (95% CI)[c], p	1.00 (ref)	0.80 (0.71-0.90), <0.001	0.79 (0.64-0.97), 0.022	0.73 (0.59-0.90), 0.003	0.70 (0.57-0.87), 0.002
Heart diseases					
No. of participants (n)	7234	415	127	120	126
Events (n)	964	65	18	21	19
Follow-up (person-years)	28269.4	2183.4	649.9	573.9	666.2
Event rate (95% CI)[b]	3.4 (3.2-3.6)	3.0 (2.3-3.7)	2.8 (1.5-4.0)	3.7 (2.1-5.2)	2.9 (1.6-4.1)
Adjusted HR (95% CI)[c], p	1.00 (ref)	0.81 (0.63-1.04), 0.104	0.79 (0.49-1.26), 0.313	0.93 (0.6-1.43), 0.736	0.77 (0.48-1.21), 0.258

[a] Multiple imputation was performed by chained equations to create 10 datasets, of which the resultant model estimates for each were combined using Rubin`s rules. The present sample size was a little different from the sample size in flow chart, which was caused by the partial overlap between the lost participants and the participants with missing baseline data.

[b] per 100 person-years.

[c] For all-cause mortality: adjustment with sex, age, education, marital status, residence, co-residence, current smoking, current drinking, current regular exercise, regular intake of foods, comorbidities (as shown in Table 1), and ADL disability. For heart diseases: adjustment with sex, age, education, marital status, residence, co-residence, current smoking, current drinking, current regular exercise, regular intake of foods, comorbidities (as shown in eTable 3), and ADL disability.

Abbreviations: CI = confidence interval, HR = hazard ratio.

Supplementary Table 9. Association between changes of social activity frequency and outcomes in the PSM sample[a]

	Never	Switching to sometimes	Switching to once a month	Switching to once a week	Switching to everyday
All-cause mortality					
No. of participants (n)	2044	545	167	149	177
Events (n)	1035	266	87	80	76
Follow-up (person-years)	10487.7	3014.9	944.3	773.8	1031.2
Event rate (95% CI)[b]	9.9 (9.3-10.4)	8.8 (7.8-9.8)	9.2 (7.4-11.1)	10.3 (8.2-12.5)	7.4 (5.8-9.0)
Adjusted HR (95% CI)[c], p	1.00 (ref)	0.85 (0.74-0.97), 0.017	0.81 (0.65-1.00), 0.055	0.75 (0.60-0.94), 0.014	0.74 (0.59-0.94), 0.013
Heart diseases					
No. of participants (n)	1470	397	121	114	119
Events (n)	261	64	18	20	18
Follow-up (person-years)	7075.4	2085.3	621.9	543.2	633.4
Event rate (95% CI)[b]	3.7 (3.2-4.1)	3.1 (2.3-3.8)	2.9 (1.6-4.2)	3.7 (2.1-5.3)	2.8 (1.5-4.1)
Adjusted HR (95% CI)[c], p	1.00 (ref)	0.81 (0.61-1.07), 0.131	0.80 (0.49-1.29), 0.351	0.86 (0.55-1.37), 0.531	0.72 (0.44-1.17), 0.183

[a] Propensity score distributional overlap and ASD are shown in eFigures 3 and 4.

[b] per 100 person-years.

[c] To eliminate the risk of insufficient covariate balance, we further adjusted for baseline covariates in the analyses (called the "doubly robust" method). For all-cause mortality: adjustment with sex, age, education, marital status, residence, co-residence, current smoking, current drinking, current regular exercise, regular intake of foods, comorbidities (as shown in Table 1), and ADL disability. For heart diseases: adjustment with sex, age, education, marital status, residence, co-residence, current smoking, current drinking, current regular exercise, regular intake of foods, comorbidities (as shown in eTable 3), and ADL disability.

Abbreviations: ASD = Absolute standardized mean differences, CI = confidence interval, PSM = propensity score matching, HR = hazard ratio.

Supplementary Table 10. Association between changes of social activity frequency and incidental heart diseases, accounting for competing risk of all-cause mortality

	Never	Switching to sometimes	Switching to once a month	Switching to once a week	Switching to everyday
No. of participants (n)	6743	397	121	115	120
Heart diseases (n)	915	64	18	20	18
All-cause mortality (n)[a]	4000	170	55	51	46
Adjusted HR (95% CI)[b], p	1.00 (ref)	0.91 (0.71-1.17), 0.450	0.89 (0.56-1.42), 0.630	1.11 (0.71-1.74), 0.650	0.89 (0.56-1.41), 0.620

[a] All-cause mortality coded as event of competing risk.

[b] For heart diseases: adjustment with sex, age, education, marital status, residence, co-residence, current smoking, current drinking, current regular exercise, regular intake of foods, comorbidities (as shown in eTable 3), and ADL disability.

Abbreviations: CI = confidence interval, HR = hazard ratio.

Supplementary Table 11. Association between changes of social activity frequency and outcomes, with age as the time scale

	Never	Switching to sometimes	Switching to once a month	Switching to once a week	Switching to everyday
All-cause mortality					
No. of participants (n)	8943	546	167	151	177
Events (n)	5897	266	87	81	76
Follow-up (person-years)	40346.1	3251.1	1003.7	844.6	1100.8
Event rate (95% CI)[a]	14.6 (14.3-15.0)	8.2 (7.2-9.1)	8.7 (6.9-10.4)	9.6 (7.6-11.6)	6.9 (5.4-8.4)
Adjusted HR (95% CI)[b], p	1.00 (ref)	0.79 (0.69-0.89), <0.001	0.78 (0.63-0.97), 0.023	0.71 (0.57-0.88), 0.002	0.70 (0.55-0.87), 0.002
Heart diseases					
No. of participants (n)	6743	397	121	115	120
Events (n)	915	64	18	20	18
Follow-up (person-years)	27715.3	2227.5	652.0	591.8	681.5
Event rate (95% CI)[a]	3.3 (3.1-3.5)	2.9 (2.2-3.6)	2.8 (1.5-4.0)	3.4 (1.9-4.8)	2.6 (1.4-3.8)
Adjusted HR (95% CI)[b], p	1.00 (ref)	0.86 (0.67-1.12), 0.266	0.89 (0.56-1.42), 0.623	0.95 (0.61-1.48), 0.808	0.79 (0.50-1.27), 0.333

[a] per 100 person-years.

[b] For all-cause mortality: adjustment with sex, age (as the time scale), education, marital status, residence, co-residence, current smoking, current drinking, current regular exercise, regular intake of foods, comorbidities (as shown in Table 1), and ADL disability. For heart diseases: adjustment with sex, age (as the time scale), education, marital status, residence, co-residence, current smoking, current drinking, current regular exercise, regular intake of foods, comorbidities (as shown in eTable 3), and ADL disability.

Abbreviations: CI = confidence interval, HR = hazard ratio.

Supplementary Table 12. Association between changes of social activity frequency and outcomes, further controlling provinces

	Never	Switching to sometimes	Switching to once a month	Switching to once a week	Switching to everyday
All-cause mortality					
No. of participants (n)	8943	546	167	151	177
Events (n)	5897	266	87	81	76
Follow-up (person-years)	38335.1	3022.0	944.3	790.1	1031.2
Event rate (95% CI)[a]	15.4 (15-15.7)	8.8 (7.8-9.8)	9.2 (7.4-11.1)	10.3 (8.1-12.4)	7.4 (5.8-9.0)
Adjusted HR (95% CI)[b], p	1.00 (ref)	0.79 (0.70-0.90), <0.001	0.76 (0.61-0.94), 0.012	0.73 (0.58-0.91), 0.005	0.70 (0.56-0.88), 0.003
Heart diseases					
No. of participants (n)	6743	397	121	115	120
Events (n)	915	64	18	20	18
Follow-up (person-years)	26641.8	2085.3	621.9	553.0	643.4
Event rate (95% CI)[a]	3.4 (3.2-3.7)	3.1 (2.3-3.8)	2.9 (1.6-4.2)	3.6 (2.1-5.2)	2.8 (1.5-4.1)
Adjusted HR (95% CI)[b], p	1.00 (ref)	0.90 (0.69-1.16), 0.408	0.83 (0.51-1.32), 0.426	0.99 (0.63-1.54), 0.948	0.70 (0.43-1.12), 0.135

[a] per 100 person-years.

[b] For all-cause mortality: adjustment with sex, age, education, marital status, residence, co-residence, current smoking, current drinking, current regular exercise, regular intake of foods, comorbidities (as shown in Table 1), ADL disability, and provinces. For heart diseases: adjustment with sex, age, education, marital status, residence, co-residence, current smoking, current drinking, current regular exercise, regular intake of foods, comorbidities (as shown in eTable 3), ADL disability, and provinces.

Abbreviations: CI = confidence interval, HR = hazard ratio.

Supplementary Table 13. E-value assessing unmeasured confoundings between changes of social activity frequency and outcomes

	Never	Switching to sometimes	Switching to once a month	Switching to once a week	Switching to everyday
All-cause mortality					
Adjusted HR (95% CI)[a], p	1.00 (ref)	0.79 (0.70-0.90), <0.001	0.78 (0.63-0.96), 0.019	0.74 (0.59-0.92), 0.006	0.70 (0.56-0.88), 0.002
E-value	NA	1.64	1.66	1.76	1.88
Heart diseases					
Adjusted HR (95% CI)[a], p	1.00 (ref)	0.83 (0.65-1.08), 0.170	0.82 (0.51-1.31), 0.412	0.91 (0.58-1.42), 0.675	0.75 (0.47-1.20), 0.227
E-value	NA	1.69	1.74	1.43	1.99

[a] For all-cause mortality: adjustment with sex, age, education, marital status, residence, co-residence, current smoking, current drinking, current regular exercise, regular intake of foods, comorbidities (as shown in Table 1), and ADL disability. For heart diseases: adjustment with sex, age, education, marital status, residence, co-residence, current smoking, current drinking, current regular exercise, regular intake of foods, comorbidities (as shown in eTable 3), and ADL disability.

Other detailed descriptions about E-values can be found in the table below on this page. Abbreviations: CI = confidence interval, HR = hazard ratio.

Supplementary Figure 1. Stratified analyses by potential modifiers of the association between changes of social activity frequency and all-cause mortality

Changes of social activity frequency	Events (rates) in subgroups	Adjusted HR (95% CI)	Events (rates) in subgroups	Adjusted HR (95% CI)	p for interaction
	Sex: male		Sex: female		
Never	2322 (64.52%)	1.00 (ref)	3575 (66.90%)	1.00 (ref)	
Once a month, sometimes	213 (53.25%)	0.86 (0.74-0.99)	140 (44.73%)	0.70 (0.59-0.83)	0.222
Everyday, once a week	91 (50.00%)	0.70 (0.57-0.87)	66 (45.21%)	0.73 (0.57-0.93)	
	Age: < 84 years		Age: ≥ 84 years		
Never	1708 (42.19%)	1.00 (ref)	4189 (85.58%)	1.00 (ref)	
Once a month, sometimes	194 (39.03%)	0.86 (0.74-1.00)	159 (73.61%)	0.68 (0.58-0.80)	0.043
Everyday, once a week	74 (32.31%)	0.72 (0.57-0.91)	83 (83.84%)	0.68 (0.55-0.85)	
	Education: no school		Education: 1 year or more		
Never	4162 (70.49%)	1.00 (ref)	1735 (57.09%)	1.00 (ref)	
Once a month, sometimes	183 (57.19%)	0.78 (0.67-0.91)	170 (43.26%)	0.83 (0.70-0.97)	0.837
Everyday, once a week	83 (56.08%)	0.68 (0.55-0.85)	74 (41.11%)	0.78 (0.62-0.99)	
	Marital status: not in marriage		Marital status: in marriage		
Never	4296 (75.97%)	1.00 (ref)	1601 (48.69%)	1.00 (ref)	
Once a month, sometimes	200 (62.31%)	0.76 (0.65-0.87)	153 (39.03%)	0.86 (0.73-1.02)	0.861
Everyday, once a week	87 (59.18%)	0.71 (0.57-0.88)	70 (38.67%)	0.73 (0.58-0.93)	
	Residence: rural		Residence: urban		
Never	3912 (65.20%)	1.00 (ref)	1985 (67.45%)	1.00 (ref)	
Once a month, sometimes	201 (52.89%)	0.93 (0.80-1.07)	152 (45.65%)	0.66 (0.56-0.79)	0.004
Everyday, once a week	81 (47.09%)	0.64 (0.52-0.8)	76 (48.72%)	0.84 (0.67-1.06)	
	Co-residence: without family		Co-residence: with family		
Never	995 (66.91%)	1.00 (ref)	4902 (65.75%)	1.00 (ref)	
Once a month, sometimes	78 (64.46%)	0.84 (0.67-1.07)	275 (46.45%)	0.79 (0.70-0.90)	0.751
Everyday, once a week	29 (49.15%)	0.71 (0.49-1.03)	128 (47.58%)	0.74 (0.62-0.88)	
	Lifestyles: unhealthy		Lifestyles: healthy		
Never	4888 (65.88%)	1.00 (ref)	1009 (66.21%)	1.00 (ref)	
Once a month, sometimes	283 (51.55%)	0.82 (0.72-0.92)	70 (42.68%)	0.71 (0.55-0.91)	0.244
Everyday, once a week	119 (48.18%)	0.69 (0.58-0.83)	38 (46.91%)	0.83 (0.60-1.15)	
	Comorbidities: no		Comorbidities: yes		
Never	4091 (66.38%)	1.00 (ref)	1806 (64.96%)	1.00 (ref)	
Once a month, sometimes	241 (50.21%)	0.82 (0.72-0.94)	112 (48.07%)	0.72 (0.59-0.87)	0.170
Everyday, once a week	115 (51.57%)	0.81 (0.67-0.98)	42 (40.00%)	0.55 (0.40-0.75)	
	ADL: no		ADL: yes		
Never	4710 (62.06%)	1.00 (ref)	1187 (87.67%)	1.00 (ref)	
Once a month, sometimes	314 (47.50%)	0.83 (0.74-0.93)	39 (75.00%)	0.62 (0.44-0.86)	0.409
Everyday, once a week	140 (45.60%)	0.74 (0.63-0.88)	17 (80.95%)	0.61 (0.37-0.99)	

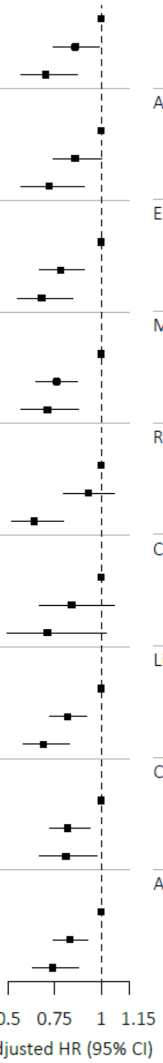

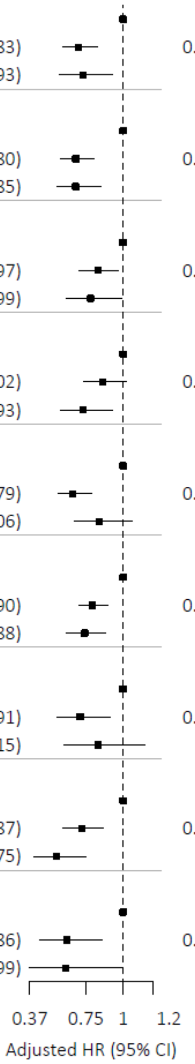

Note:

For lifestyles, a participant would be defined as “healthy” with meeting all the criterions, including current smoking (no), current drinking (no), and current regular exercise (yes); otherwise, he/she was defined as “unhealthy”.

For comorbidities, any of the diseases shown in Table 1 was present, and the participant would be defined as “yes”; otherwise, he/she was defined as “no”.

Each stratification adjusted for all factors (sex, age, education, marital status, residence, co-residence, current smoking, current drinking, current regular exercise, regular intake of foods, comorbidities [as shown in Table 1], and ADL disability) except the stratification factor itself.

Grouping criteria of continuous variables were based on the median values.

Abbreviations: ADL = activities of daily living.

Supplementary Figure 2. Stratified analyses by potential modifiers of the association between changes of social activity frequency and heart diseases

Changes of social activity frequency	Events (rates) in subgroups	Adjusted HR (95% CI)	Events (rates) in subgroups	Adjusted HR (95% CI)	p for interaction
	Sex: male		Sex: female		
Never	390 (14.21%)	1.00 (ref)	525 (13.13%)	1.00 (ref)	
Once a month, sometimes	49 (17.01%)	0.95 (0.70-1.29)	33 (14.35%)	0.71 (0.49-1.01)	0.618
Everyday, once a week	22 (16.79%)	0.84 (0.54-1.29)	16 (15.38%)	0.82 (0.49-1.35)	
	Age: < 85 years		Age: ≥ 85 years		
Never	581 (18.75%)	1.00 (ref)	334 (9.16%)	1.00 (ref)	
Once a month, sometimes	68 (17.99%)	0.85 (0.66-1.09)	14 (10.00%)	0.76 (0.44-1.32)	0.622
Everyday, once a week	31 (18.79%)	0.93 (0.65-1.35)	7 (10.00%)	0.62 (0.29-1.32)	
	Education: no school		Education: 1 year or more		
Never	575 (12.68%)	1.00 (ref)	340 (15.38%)	1.00 (ref)	
Once a month, sometimes	32 (13.11%)	0.66 (0.46-0.95)	50 (18.25%)	1.00 (0.74-1.35)	0.245
Everyday, once a week	16 (14.95%)	0.76 (0.46-1.26)	22 (17.19%)	0.88 (0.56-1.36)	
	Marital status: not in marriage		Marital status: in marriage		
Never	498 (11.45%)	1.00 (ref)	417 (17.43%)	1.00 (ref)	
Once a month, sometimes	23 (9.79%)	0.54 (0.35-0.83)	59 (20.85%)	1.10 (0.83-1.45)	0.019
Everyday, once a week	18 (15.79%)	0.92 (0.57-1.48)	20 (16.53%)	0.76 (0.48-1.20)	
	Residence: rural		Residence: urban		
Never	600 (13.00%)	1.00 (ref)	315 (14.80%)	1.00 (ref)	
Once a month, sometimes	41 (13.67%)	0.78 (0.57-1.07)	41 (18.81%)	0.88 (0.63-1.23)	0.126
Everyday, once a week	15 (11.63%)	0.63 (0.38-1.06)	23 (21.70%)	1.02 (0.66-1.58)	
	Co-residence: without family		Co-residence: with family		
Never	315 (14.8%)	1.00 (ref)	600 (13.00%)	1.00 (ref)	
Once a month, sometimes	41 (18.81%)	0.53 (0.27-1.05)	41 (13.67%)	0.91 (0.71-1.16)	0.228
Everyday, once a week	23 (21.70%)	0.97 (0.47-2.00)	15 (11.63%)	0.80 (0.56-1.16)	
	Lifestyles: unhealthy		Lifestyles: healthy		
Never	755 (13.37%)	1.00 (ref)	160 (14.60%)	1.00 (ref)	
Once a month, sometimes	66 (15.94%)	0.87 (0.67-1.12)	16 (15.38%)	0.75 (0.44-1.29)	0.781
Everyday, once a week	28 (15.56%)	0.81 (0.55-1.18)	10 (18.18%)	1.00 (0.52-1.95)	
	Comorbidities: no		Comorbidities: yes		
Never	617 (12.24%)	1.00 (ref)	298 (17.52%)	1.00 (ref)	
Once a month, sometimes	54 (14.06%)	0.79 (0.59-1.05)	28 (20.90%)	0.97 (0.65-1.44)	0.112
Everyday, once a week	21 (11.73%)	0.69 (0.45-1.07)	17 (30.36%)	1.15 (0.69-1.90)	
	ADL: no		ADL: yes		
Never	791 (13.80%)	1.00 (ref)	124 (12.24%)	1.00 (ref)	
Once a month, sometimes	77 (15.78%)	0.83 (0.65-1.05)	5 (16.67%)	0.71 (0.25-1.95)	0.689
Everyday, once a week	36 (16.29%)	0.86 (0.62-1.21)	2 (14.29%)	0.43 (0.10-1.83)	

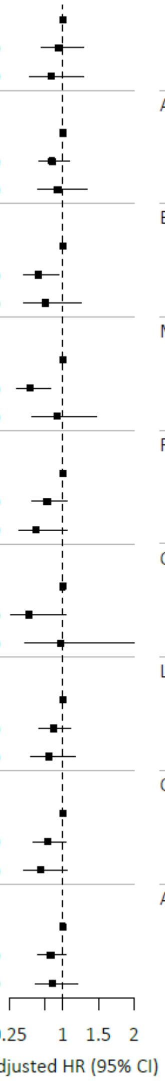

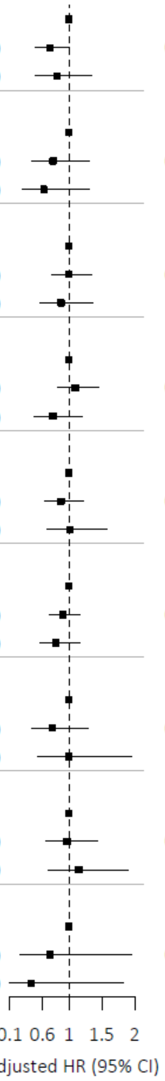

Note:

For lifestyle, a participant would be defined as "healthy" with meeting all the criterions, including current smoking (no), current drinking (no), and current regular exercise (yes); otherwise, he/she was defined as "unhealthy".

For comorbidities, any of the diseases shown in eTable 3 was present, and the participant would be defined as "yes"; otherwise, he/she was defined as "no".

Each stratification adjusted for all factors (sex, age, education, marital status, residence, co-residence, current smoking, current drinking, current regular exercise, regular intake of foods, comorbidities [as shown in eTable 3], and ADL disability) except the stratification factor itself.

Grouping criteria of continuous variables were based on the median values.

Abbreviations: ADL = activities of daily living.

Supplementary Figure 3. Propensity score distributional overlap and ASD (for assessing risk of all-cause mortality)

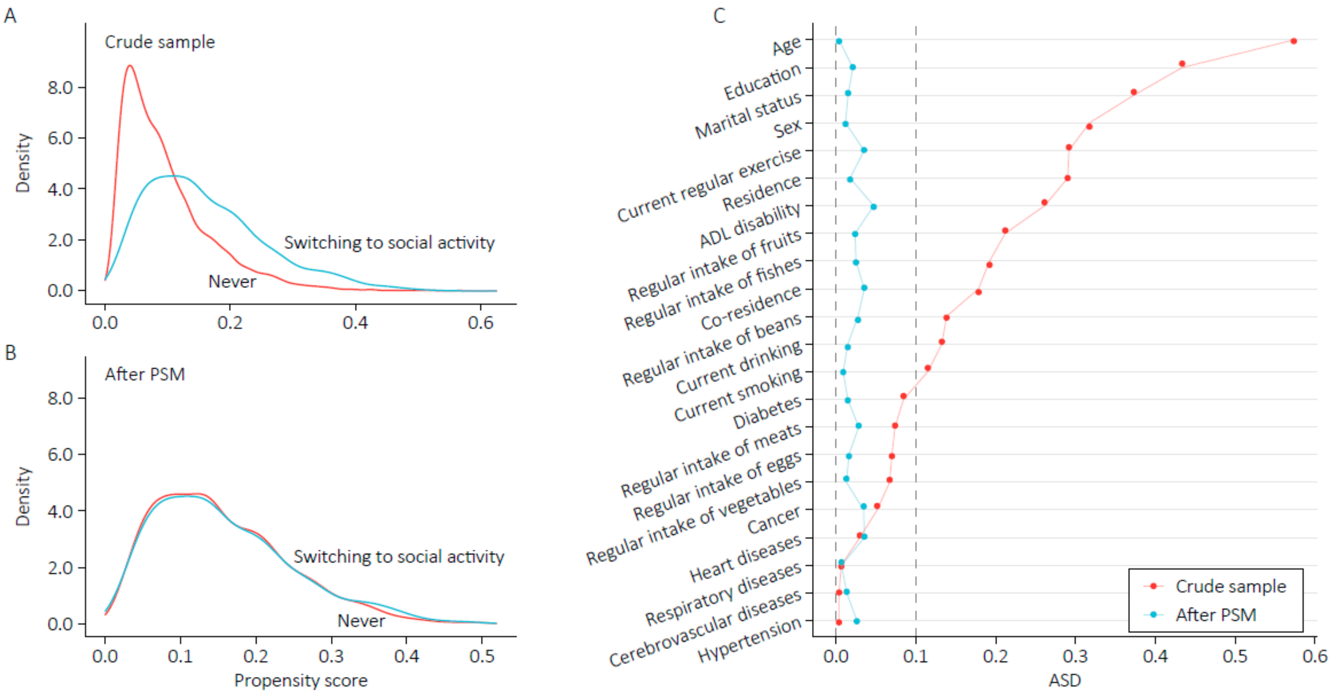

Note:
The two groups, namely never vs. switching to social activity, were matched by PSM, which was performed using the nearest neighbor matching algorithm, with a fixed caliper of 0.1 (2:1 matching, without replacement). Predicting " switching to social activity " was modeld by multivariable logistic regression analysis, and C-index was 0.716.

Figures A and B present the distributions of propensity score between the two groups in the crude and PSM samples. For intervals along the x-axis, the area under the probability density curve represents the probability of those propensity scores, and smoothing was via the kernel density estimate. Greater overlap of propensity score curves of the two groups indicates a lesser risk of confounding.

Figure C shows ASD between the two groups in the crude and PSM samples. ASD creates a uniform scaling by which imbalance in variables may be assessed; the dashed line indicates greater than 0.100 imbalance between the variable’s values, which is a commonly used metric of significant imbalance.

Abbreviations: ADL = activities of daily living, ASD = Absolute standardized mean differences, PSM = propensity score matching.

Supplementary Figure 4. Propensity score distributional overlap and ASD (for assessing risk of heart diseases)

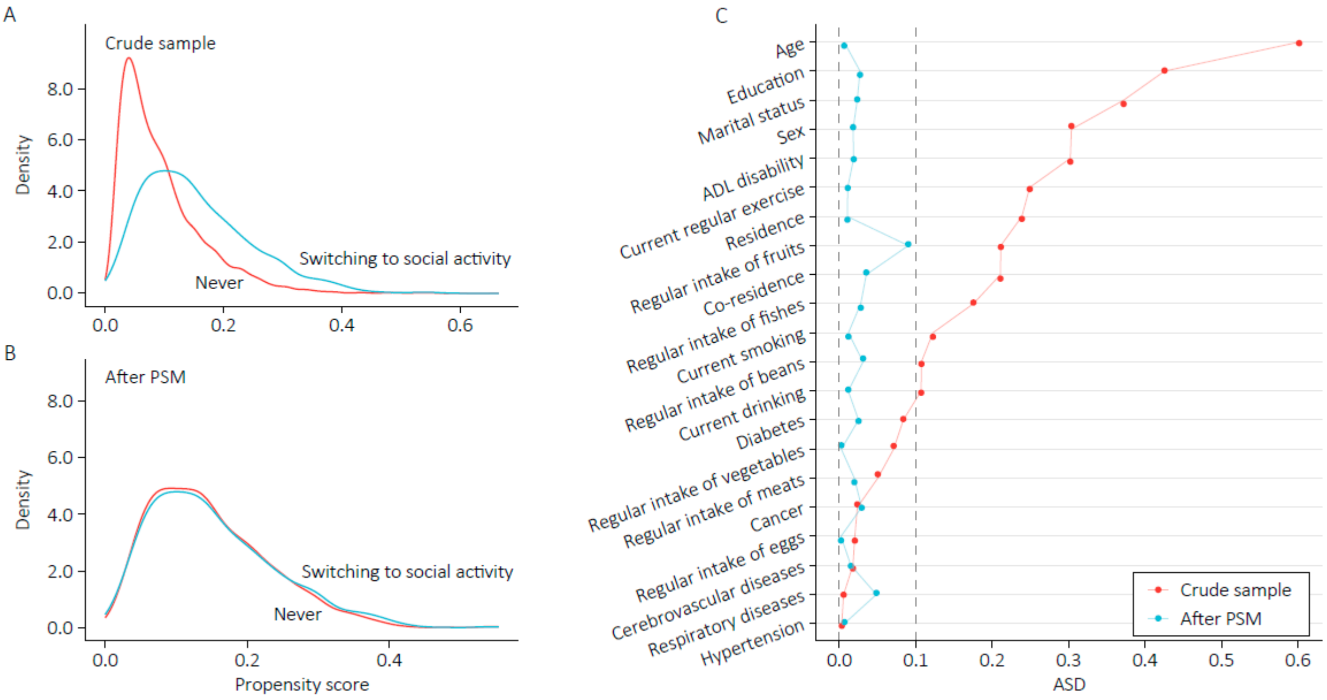

Note:

The two groups, namely never vs. switching to social activity, were matched by PSM, which was performed using the nearest neighbor matching algorithm, with a fixed caliper of 0.1 (2:1 matching, without replacement). Predicting " switching to social activity " was modeld by multivariable logistic regression analysis, and C-index was 0.717.

Figures A and B present the distributions of propensity score between the two groups in the crude and PSM samples. For intervals along the x-axis, the area under the probability density curve represents the probability of those propensity scores, and smoothing was via the kernel density estimate. Greater overlap of propensity score curves of the two groups indicates a lesser risk of confounding.

Figure C shows ASD between the two groups in the crude and PSM samples. ASD creates a uniform scaling by which imbalance in variables may be assessed; the dashed line indicates greater than 0.100 imbalance between the variable's values, which is a commonly used metric of significant imbalance.

Abbreviations: ADL = activities of daily living, ASD = Absolute standardized mean differences, PSM = propensity score matching.

Supplementary Figure 5. Cumulative incidence of heart diseases based on changes of social activity frequency (solid line) and adjusted for competing risk of all-cause mortality

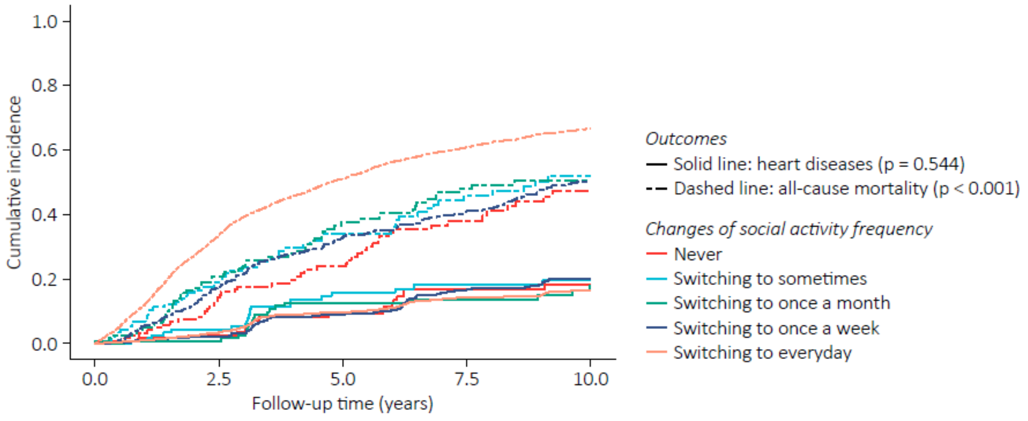

Supplement: Supplementary file 1 [file Data_Sheet_1.pdf]
